# Ion release dynamics of bioactive resin cement under variable pH conditions

**DOI:** 10.3389/froh.2025.1564838

**Published:** 2025-03-12

**Authors:** Venkata Suresh Venkataiah, Jogikalmat Krithikadatta, Kavalipurapu Venkata Teja, Deepak Mehta, Mukesh Doble

**Affiliations:** ^1^ Department of Cariology, Saveetha Dental College and Hospitals, Saveetha Institute of Medical and Technical Sciences, Saveetha University, Chennai, India; ^2^ Department of Conservative Dentistry and Endodontics, Mamata Institute of Dental Sciences, Hyderabad, India; ^3^ Department of Operative Dentistry, Tohoku Graduate School of Dentistry, Sendai, Japan

**Keywords:** dental biomaterials, remineralization, ion release, predicta bioactive self-adhesive cement, PH sensitivity, CPP-ACP paste

## Abstract

**Introduction:**

Understanding the ion release behaviour of bioactive resin cements is essential for evaluating their potential in restorative dentistry. Sustained ion release, especially at cariogenic pH levels, can enhance remineralization and prolong the longevity of dental restorations. This study investigates the influence of pH on the ion release profiles of a bioactive resin cement before and after recharging.

**Methods:**

Disk-shaped specimens (*n* = 15) of bioactive resin cement were prepared and exposed to three different pH conditions (4.5, 5.5, and 6.5) to simulate normal and cariogenic environments. Calcium ion release was quantified using atomic spectrophotometry, while fluoride and phosphate ions were analyzed using quantitative spectrophotometry. After an initial 30-day depletion phase, recharging was performed using casein phosphopeptide-amorphous calcium phosphate with fluoride (CPP-ACPF) paste.

**Results:**

In the pre-recharging phase (Stage 1), calcium ion release was more pronounced at acidic pH (4.5–5.5), particularly in the first five days. Fluoride and phosphate ions also demonstrated higher release at pH 4.5 and 5.5 compared to pH 6.5. Post-recharging (Stage 2) exhibited similar trends, emphasizing the role of regular recharge in sustaining ion availability. The absence of an initial burst release, commonly seen in other bioactive materials, suggests a distinct ion release mechanism in these resin cements.

**Conclusion:**

The findings highlight the pH-dependent release characteristics of bioactive resin cements and reinforce the importance of recharging for maintaining their therapeutic potential. The unique release kinetics observed may offer advantages in long-term remineralization strategies for dental restorations.

## 1 Introduction

Dental cements play a pivotal role in modern restorative dentistry, serving as the foundation for various clinical applications, including the placement of crowns, bridges, and orthodontic appliances (1). Resin cements form chemical bonds with dentine and enamel, offering strong adhesive properties and predictable long-term performance (1). Over the years, self-adhesive resin cements, which eliminate the need for separate adhesives and etchants, have gained popularity due to their shorter application times and ease of use (2). Beyond these technical advantages, enhancing the bioactivity of dental cements is of growing interest, particularly for remineralization and caries prevention. However, conventional resin cements exhibit limited intrinsic cariostatic effects, necessitating the development of bioactive alternatives.

Secondary caries, a leading cause of prosthetic restoration failure, has significant clinical and economic implications (3). Alenezi et al. (4) report that approximately 8.4% of restorations fail due to secondary caries, often resulting from poor oral hygiene and the lack of self-cleaning ability in restored tooth structures. A promising approach to enhancing the longevity of restorations involves using bioactive cements capable of releasing remineralizing ions. Fluoride, calcium, and phosphate are essential for enamel repair, with fluoride playing a well-established role in inhibiting demineralization and interfering with the metabolism of cariogenic bacteria (5–7).

However, conventional fluoride-based preventive strategies, such as toothpastes and mouthwashes, depend on patient compliance and require consistent application for optimal efficacy (8, 9). Similarly, calcium- and phosphate-based remineralization agents, including casein phosphopeptide-amorphous calcium phosphate fluoride (CPP-ACPF) pastes, offer potential benefits but may be influenced by factors such as salivary pH and require frequent reapplication (10–13).

Recent advancements in materials science have led to the development of “smart” biomaterials capable of sustained ion release and rechargeability (13–15). Rechargeable bioactive cements can replenish their ion reservoirs, ensuring prolonged therapeutic effects over time. Among these, Predicta Bioactive Resin Cement has emerged as a promising material due to its unique ability to release and recharge calcium, phosphate, and fluoride ions. Unlike traditional bioactive cements, Predicta Bioactive Resin Cement combines adhesive strength with ion-releasing capabilities, making it a viable option for improving restoration longevity and caries prevention.

In light of these developments, this study evaluates the ion release characteristics of Predicta Bioactive Resin Cement under different pH conditions, both before and after recharging with CPP-ACPF paste. We hypothesize that the cement will release calcium, phosphate, and fluoride ions optimally across different pH levels and that recharging with CPP-ACPF paste will significantly enhance this release. Specifically, this *in vitro* investigation aims to elucidate the patterns of ion release from Predicta Bioactive Resin Cement and assess its potential for long-term remineralization. The findings of this study may inform clinical decision-making by highlighting the advantages of smart biomaterials in restorative dentistry.

## 2 Materials and methods

A review of the literature was undertaken to identify optimal methodological details for conducting an *in vitro* study to assess ion release from dental bioactive resin cements. The material used in this study was Predicta Bioactive Resin Cement (Parkell, USA), a commercially available bioactive resin cement known for its calcium, fluoride, and phosphate ion release capabilities (Supplemental Table 1). The detailed composition and applications of this cement were derived from manufacturer specifications and existing literature. The specimens in this study were composed entirely of bioactive resin cement and did not involve enamel or dentin. Therefore, no surface pretreatment was performed.

A protocol ensuing from this methodological review was used to conduct a two-stage *in vitro* study. In stage 1 of this study, we assessed the release of calcium, fluoride and phosphate ions at pH values of 4.5, 5.5 and 6.5. In stage 2, the samples were recharged with casein phosphorous-peptide-amorphous calcium phosphate containing fluoride paste (CPP-ACPF paste; Tooth Mousse, GC, Tokyo, Japan) and the release of calcium, fluoride and phosphate ions were re-assessed at pH values of 4.5, 5.5 and 6.5. Ion release was analyzed using spectrophotometry and was conducted at Indian Institute of Technology, Chennai.

### 2.1 Preparation of test samples

In this study, 15 disk specimens of Predicta Bioactive Resin Cement were carefully placed into molds measuring 2 mm × 2 mm × 10 mm made of condensational silicone material. Each layer of these molds, totaling 1 mm, underwent a 40-second curing process using a halogen light curing unit (Optilux 501, Kerr, USA) with a power density of 550 mW/cm^2^. Subsequently, the disk specimens were extracted from the molds and subjected to an additional 40 s curing session from a perpendicular distance of 1 mm to ensure complete polymerization. The disk specimens were divided into three groups (*n* = 5 per group) based on the pH of the immersion solution: group A (pH 4.5), group B (pH 5.5), and group C (pH 6.5). The pH of the immersion buffer solutions, consisting of citric acid and deionized distilled water, was adjusted using sodium hydroxide or hydrochloric acid and was carefully monitored throughout the study using a calibrated pH meter to ensure consistency within ±0.1 of the target values. Fresh buffer solutions were prepared daily to maintain stable pH conditions.

### 2.2 Justification for pH selection

The pH values selected for this study (4.5, 5.5, and 6.5) simulate various oral conditions encountered in clinical practice. A pH of 4.5 represents an acidic oral environment, such as those observed during cariogenic bacterial activity and dietary acid exposure. A pH of 5.5 corresponds to the critical pH of enamel, below which demineralization occurs, making it a clinically relevant threshold for evaluating remineralization potential. A pH of 6.5 represents near-neutral salivary conditions, mimicking a non-pathological oral environment where bioactive materials may still contribute to sustained remineralization. These pH values were chosen to assess the bioactive cement's ion release capabilities under varying degrees of acidic challenge, reflecting real-world conditions.

### 2.3 Justification for CPP-ACPF as the recharging agent

The selection of casein phosphopeptide-amorphous calcium phosphate fluoride (CPP-ACPF) paste (Tooth Mousse, GC, Tokyo, Japan) as the recharging agent was based on its well-documented remineralization potential. CPP-ACPF stabilizes calcium and phosphate ions in an amorphous form, facilitating their bioavailability for enamel and dentin remineralization (10). Additionally, CPP-ACPF has demonstrated superior ion release compared to conventional fluoride gels, as it provides a sustained supply of calcium, phosphate, and fluoride ions (11, 12). The choice of CPP-ACPF was made to mimic clinically available remineralization agents and evaluate their ability to enhance the recharge capacity of Predicta Bioactive Resin Cement.

### 2.4 Ion release measurements in stage 1

In stage 1, the disk specimens were immersed in 15 ml of their respective buffer solutions. Ion release measurements were conducted on days 1, 3, 7, 14, and 21. On each of these days, 5 ml of the solution was extracted for analysis, and the remaining solution was discarded. Fresh buffer solutions were prepared, and the samples were re-immersed after each extraction. pH verification was performed before each immersion to ensure that any potential fluctuations due to ion exchange were corrected, maintaining the experimental conditions. On day 21, the samples were immersed in fresh buffer solutions and left undisturbed for an additional 30 days to ensure “zero-ion release”. The confirmation of “zero-ion release” was performed using spectrophotometry, ensuring that no detectable calcium, phosphate, or fluoride ions were released into the buffer solution.

### 2.5 Ion release measurements in stage 2

After the 30-day period to confirm “zero-ion release”, the samples were recharged by immersing them in a solution containing 1.7 g of CPP-ACPF paste dissolved in 5 ml of deionized water. The specimens were gently stirred in a vortex machine for 1 min and left undisturbed for 30 min to mimic clinical conditions. The specimens were then rinsed with deionized water to remove surface ions and re-immersed in fresh buffer solutions. Ion release measurements were conducted on post-recharge days 1, 3, 7, and 14, with 5 ml of the solution extracted for analysis on each day. Specimens were recharged twice daily, simulating morning and evening mouth rinses.

### 2.6 Spectroscopic analysis

In this study, calcium analysis was performed using atomic absorption spectroscopy, phosphate analysis was performed using spectrophotometer and fluoride measurements were done using an ion selective electrode.

### 2.7 Statistical analysis

The sample size for this study was determined using power analysis, considering an effect size of 0.5 (medium effect size based on prior studies on ion release from bioactive materials), a significance level of 0.05, and a power of 0.8. These parameters were chosen to ensure adequate sensitivity in detecting statistically significant differences in ion release across different pH levels and time points while minimizing the risk of Type I and Type II errors. The calculated sample size of 15 specimens per group provides sufficient confidence to support the study's findings within an acceptable margin of error. The collected data was organized into tables, and descriptive analyses were carried out to calculate the mean and standard deviation of ion release on each test day at pH values of 4.5, 5.5, and 6.5. Subsequently, a two-way analysis of variance (ANOVA) was conducted to detect any interactions. For significant interactions, a one-way ANOVA and *post-hoc* Tukey test was performed. Prior to conducting ANOVA, the assumptions of normality and homogeneity of variance were assessed using the Shapiro–Wilk test and Levene's test, respectively. Data transformation was considered if assumptions were violated. A significance level of *p* < 0.05 was applied. All statistical analyses were executed using SPSS (IBM SPSS Statistics for Windows; Version 22).

## 3 Results

### 3.1 Ion release in stage 1 of the study

**Figure 1:**
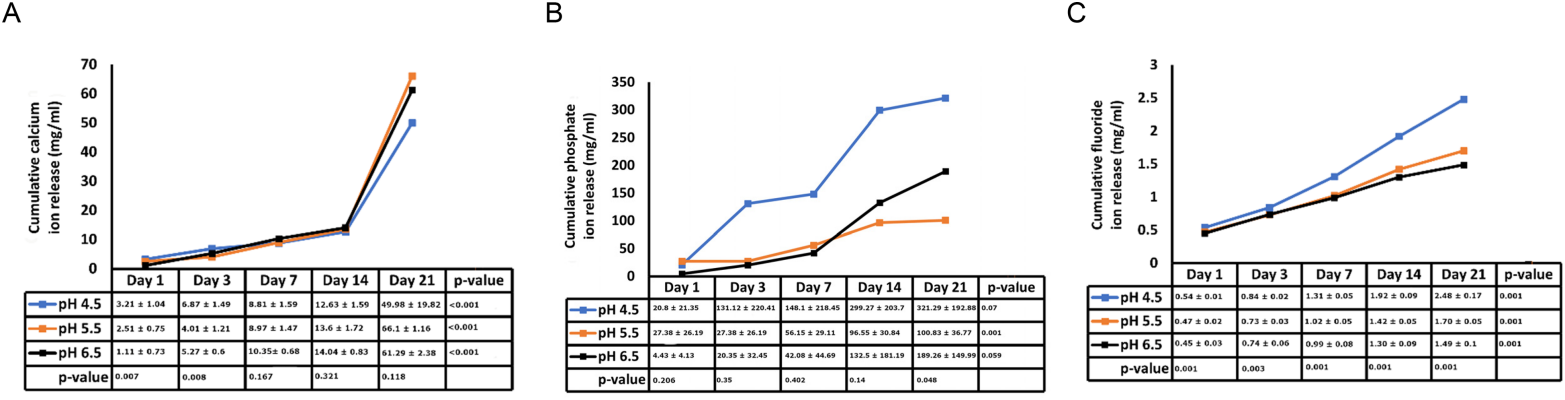
Pre- recharge Ion release. Cumulative initial ion release (pre-recharge) of calcium **(panel A)**, phosphate **(panel B)** and fluoride **(panel C)** ions at pH values of 4.5, 5.5 and 6.5 at days 1, 3, 7, 14 and 21.

Figure 1 illustrates the average initial (pre-recharge) release of calcium, phosphate and fluoride ions at pH values of 4.5, 5.5, and 6.5 on days 1, 3, 7, 14, and 21. The results of the two-way ANOVA for the initial calcium ion release revealed no significant interaction between day of analysis (time) and pH (*p* = 1.000). While, not significantly affected by pH (*p* = 0.985), a trend favoring calcium ion release at pH rage of 4.5 to 5.5 was evident, especially on days 1 and 3 (Figure 1A). Table 1 provides the mean differences in calcium ion measurements on days 1, 3, 7, 14, and 21 and is indicative of better calcium ion release during the initial phases as compared to later phases. With respect to fluoride ion release in the pre-recharge phase, results of the two-way ANOVA indicated significant interaction between day of analysis (time) and pH (*p* < 0.001). Thus, a one-way ANOVA was performed with Tukey test for *post-hoc* comparisons for the effects of day of analysis (Table 2) and pH (Table 3) on cumulative fluoride ion release. Overall, the results indicate that pH values of 4.5 and 5.5 were associated with better fluoride release as compared to pH 6.5. Tables 4, 5 present the results of effects of day of analysis and pH on phosphate ion release, respectively. Phosphate ion release was better in the earlier days during both the pre-recharge phases (Table 4). Furthermore, phosphate ion release was better with pH 4.5 as compared to pH 5.5 and 6.5 (Table 5).

**Table 1:** Mean differences in calcium ion release in pre and post-recharge phases.

Pre-recharge phase				Post-recharge phase			
(I) Day of analysis	(J) Day of analysis	Mean difference (I-J) ^a^	*p* -value	(I) Day of analysis	(J) Day of analysis	Mean difference (I-J) ^a^	*p* -value
Day 1	Day 3	−66.8698	0.828	Day 1	Day 7	−67.5898	0.619
	Day 7	−135.9398	0.204		Day 14	−135.7511	0.074
	Day 14	−207.4742	**0**.**011**		Day 21	−206.5904	**0**.**002**
	Day 21	−256.8962	**0**.**001**	Day 7	Day 1	67.5898	0.619
Day 3	Day 1	66.8698	0.828		Day 14	−68.1613	0.612
	Day 7	−69.0700	0.811		Day 21	−139.0007	0.064
	Day 14	−140.6044	0.176	Day 14	Day 1	135.7511	0.074
	Day 21	−190.0264	**0**.**025**		Day 7	68.1613	0.612
Day 7	Day 1	135.9398	0.204		Day 21	−70.8393	0.582
	Day 3	69.0700	0.811	Day 21	Day 1	206.5904^a^	**0**.**002**
	Day 14	−71.5344	0.790		Day 7	139.0007	**0**.**064**
	Day 21	−120.9564	0.314		Day 14	70.8393	0.582
Day 14	Day 1	207.4742	**0**.**011**				
	Day 3	140.6044	0.176				
	Day 7	71.5344	0.790				
	Day 21	−49.4220	0.936				
Day 21	Day 1	256.8962	**0**.**001**				
	Day 3	190.0264	**0**.**025**				
	Day 7	120.9564	0.314				
	Day 14	49.4220	0.936				

Computed using ANOVA and TUKEY adjustment.

Mean differences in calcium ion release were calculated based on data collected from 5 ml solutions extracted on pre-recharge and post-recharge days. The pre-recharge phase includes days 1, 3, 7, 14, and 21.

Bold values indicate statistically significant differences (*p* < 0.05) as determined by ANOVA and post-hoc Tukey test.

^a^ I-J: Mean difference; Negative difference favors the group in column (i) and positive difference favors the group in column (J).

**Table 2:** Mean differences in fluoride ion release in pre and post-recharge phases.

Pre-recharge phase				Post-recharge phase			
(I) Day of analysis	(J) Day of analysis	Mean difference (I-J) ^a^	*p* -value	(I) Day of analysis	(J) Day of analysis	Mean difference (I-J) ^a^	*p* -value
Day 1	Day 3	−0.28333	0.026	Day 1	Day 7	−0.30400	**0**.**015**
	Day 7	−0.62067	**<0**.**001**		Day 14	−0.56733	**<0**.**001**
	Day 14	−1.06133	**<0**.**001**		Day 21	−0.82000	**<0**.**001**
	Day 21	−1.40533	**<0**.**001**	Day 7	Day 1	0.30400	**0**.**015**
Day 3	Day 1	.28333	**0**.**026**		Day 14	−0.26333	**0**.**045**
	Day 7	-.33733	**0**.**005**		Day 21	−0.51600	**<0**.**001**
	Day 14	-.77800	**<0**.**001**	Day 14	Day 1	0.56733	**<0**.**001**
	Day 21	−1.12200	**<0**.**001**		Day 7	0.26333	**0**.**045**
Day 7	Day 1	.62067	**<0**.**001**		Day 21	−0.25267	0.058
	Day 3	.33733	**0**.**005**	Day 21	Day 1	0.82000	**<0**.**001**
	Day 14	−0.44067	**<0**.**001**		Day 7	0.51600	**<0**.**001**
	Day 21	−0.78467	**<0**.**001**		Day 14	0.25267	0.058
Day 14	Day 1	1.06133	**<0**.**001**				
	Day 3	0.77800	**<0**.**001**				
	Day 7	0.44067	**<0**.**001**				
	Day 21	−0.34400	**0**.**004**				
Day 21	Day 1	1.40533	**<0**.**001**				
	Day 3	1.12200	**<0**.**001**				
	Day 7	0.78467	**<0**.**001**				
	Day 14	0.34400	0.004				

Computed using ANOVA and TUKEY adjustment.

Mean differences in calcium ion release were calculated based on data collected from 5 ml solutions extracted on pre-recharge and post-recharge days. The pre-recharge phase includes days 1, 3, 7, 14, and 21.

Bold values indicate statistically significant differences (*p* < 0.05) as determined by ANOVA and post-hoc Tukey test.

^a^ I-J: Mean difference; Negative difference favors the group in column (i) and positive difference favors the group in column (J).

**Table 3:** pH-dependence of fluoride ion release in pre and post-recharge phases.

Pre-recharge phase				Post-recharge phase			
(I) pH	(J) pH	Mean Difference (I-J)^a^	*p*-value	(I) pH	(J) pH	Mean Difference (I-J)^a^	*p*-value
4.5	5.5	0.35240	0.064	4.5	5.5	0.37000	**0**.**003**
	6.5	0.42640	0.019		6.5	0.53200	**<0**.**001**
5.5	4.5	−0.35240	0.064	5.5	4.5	−0.37000	**0**.**003**
	6.5	0.07400	0.881		6.5	0.16200	0.297
6.5	4.5	−0.42640	0.019	6.5	4.5	−0.53200	**>0**.**001**
	5.5	−0.07400	0.881		5.5	−0.16200	0.297

Computed using ANOVA and TUKEY adjustment.

Data were collected from 5 ml solutions on pre-recharge and post-recharge days.

Bold values indicate statistically significant differences (*p* < 0.05) as determined by ANOVA and post-hoc Tukey test.

^a^ I-J: Mean difference; Negative difference favors the group in column (i) and positive difference favors the group in column (J).

**Table 4:** Mean differences in phosphate ion release in pre and post-recharge phases.

Pre-recharge phase				Post-recharge phase			
(I) Day of analysis	(J) Day of analysis	Mean difference (I-J)^a^	*p*-value	(I) Day of analysis	(J) Day of analysis	Mean difference (I-J)^a^	*p*-value
Day 1	Day 3	−42.07600	0.916	Day 1	Day 7	−8.77267	0.960
	Day 7	−64.56933	0.695		Day 14	−94.35200	**<**.**001**
	Day 14	−158.56400	**0**.**018**		Day 21	−152.23933	**<**.**001**
	Day 21	−196.25400	**0**.**002**	Day 7	Day 1	8.77267	0.960
Day 3	Day 1	42.07600	0.916		Day 14	−85.57933	**<**.**001**
	Day 7	−22.49333	0.991		Day 21	−143.46667	**<**.**001**
	Day 14	−116.48800	0.146	Day 14	Day 1	94.35200	**<**.**001**
	Day 21	−154.17800	**0**.**023**		Day 7	85.57933	**<**.**001**
Day 7	Day 1	64.56933	0.695		Day 21	−57.88733	**0**.**010**
	Day 3	22.49333	0.991	Day 21	Day 1	152.23933	**<**.**001**
	Day 14	−93.99467	0.335		Day 7	143.46667	**<**.**001**
	Day 21	−131.68467	0.074		Day 14	57.88733	**0**.**010**
Day 14	Day 1	158.56400	**0**.**018**				
	Day 3	116.48800	0.146				
	Day 7	93.99467	0.335				
	Day 21	−37.69000	0.942				
Day 21	Day 1	196.25400	**0**.**002**				
	Day 3	154.17800	**0**.**023**				
	Day 7	131.68467	0.074				
	Day 14	37.69000	0.942				

Computed using ANOVA and TUKEY adjustment.

Data were collected from 5 ml solutions on pre-recharge and post-recharge days.

Bold values indicate statistically significant differences (*p* < 0.05) as determined by ANOVA and post-hoc Tukey test.

^a^ I-J: Mean difference; Negative difference favors the group in column (i) and positive difference favors the group in column (J).

**Table 5:** pH-dependence of phosphate ion release in pre and post-recharge phases.

Pre-recharge phase				Post-recharge phase			
(I) Day of analysis	(J) Day of analysis	Mean Difference (I-J)	*p* -value	(I) Day of analysis	(J) Day of analysis	Mean difference (I-J)	*p* -value
4.5	5.5	128.45720^a^	**0**.**006**	4.5	5.5	38.32800	0.281
	6.5	112.39200^a^	**0**.**018**		6.5	10.71050	0.903
5.5	4.5	−128.45720^a^	**0**.**006**	5.5	4.5	−38.32800	0.281
	6.5	−16.06520	0.916		6.5	−27.61750	0.513
6.5	4.5	−112.39200^a^	**0**.**018**	6.5	4.5	−10.71050	0.903
	5.5	16.06520	0.916		5.5	27.61750	0.513

Computed using ANOVA and TUKEY adjustment.

Data were collected from 5 ml solutions on pre-recharge and post-recharge days.

Bold values indicate statistically significant differences (*p* < 0.05) as determined by ANOVA and post-hoc Tukey test.

^a^ I-J: Mean difference; Negative difference favors the group in column (i) and positive difference favors the group in column (J).

### 3.2 Post-recharge ion release in stage 2 of the study

**Figure 2:**
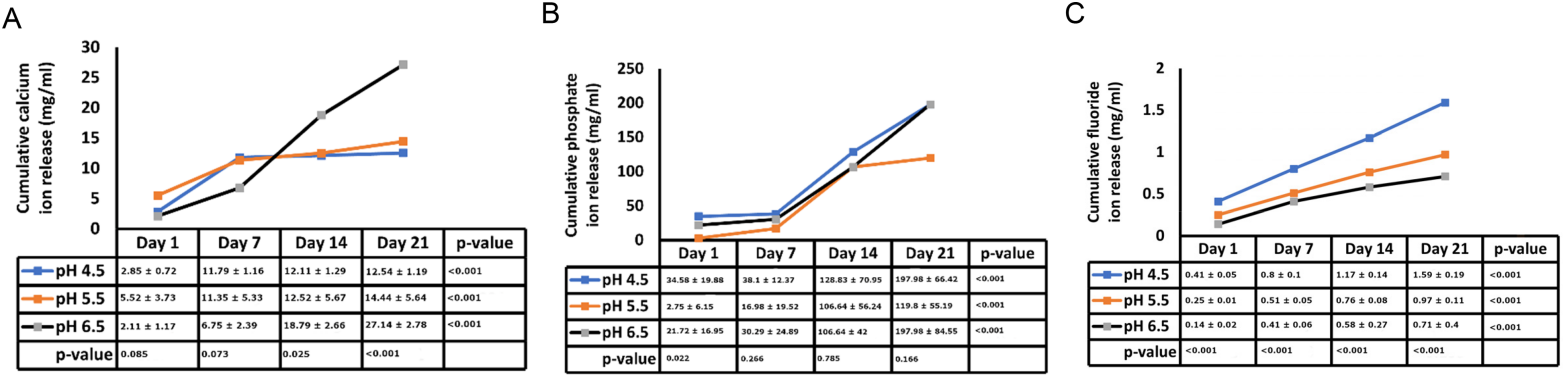
Post-recharge ion release. Cumulative post-recharge ion release of calcium **(panel A)**, phosphate **(panel B)** and fluoride **(panel C)** ions at pH values of 4.5, 5.5 and 6.5 at days 1, 3, 7, 14 and 21.

Figure 2 illustrates the average initial release of calcium, phosphate, and fluoride ions at pH levels of 4.5, 5.5, and 6.5 on days 1, 3, 7, 14, and 21. The two-way ANOVA results for initial calcium ion release did not show a significant interaction between the day of analysis (time) and pH (*p* = 1.000). However, there was a trend indicating a tendency for calcium ion release at pH levels ranging from 4.5 to 5.5, particularly noticeable on days 1 and 3 (see Figure 2A). Table 1 presents the mean differences in calcium ion measurements on days 1, 3, 14, and 21, suggesting better calcium ion release during the initial phases compared to later stages. In the pre-recharge phase, the two-way ANOVA results for fluoride ion release indicated a significant interaction between the day of analysis (time) and pH (*p* < 0.001). Therefore, a one-way ANOVA was conducted, followed by Tukey tests for *post-hoc* comparisons to assess the effects of the day of analysis (see Table 2) and pH (see Table 3) on cumulative fluoride ion release. Consistent with the pre-recharge phase, the findings suggest that pH levels of 4.5 and 5.5 were associated with better fluoride release compared to pH 6.5. Regarding phosphate ion release, it was more pronounced during the earlier days of the post-recharge phase (see Table 4). Additionally, phosphate ion release was higher at pH 4.5 compared to pH 5.5 and 6.5 (see Table 4). The ion release patterns observed in both pre and post-recharge phases were similar. Furthermore, the better ion release characteristics observed closer to recharge time points underscore the importance of regular recharge.

## 4 Discussion

Existing evidence underscores the central role of pH levels in the oral environment in the development of caries. Typically, a local plaque pH above 6 is considered “caries-safe”, a pH range of 5.5 to 6 is labeled “potentially cariogenic”, and the pH range of 4 to 5.5 is deemed highly cariogenic (16, 17). In our investigation, we observed substantial release of calcium, fluoride and phosphate ions not only at the “caries-safe” pH of 6.5 but also within the cariogenic pH ranges of 4.5 and 5.5. These observations support the remineralizing potential of bioactive resin cement, particularly in cariogenic conditions. These findings align with a study by Xu et al., which reported significant increase in calcium and phosphate release at cariogenic pH with smart calcium phosphate composites (14). In a study by Mazzaoui et al., an initial burst release of ions due to superficial dissolution of the material was described, which decelerated over time with a subsequent decrease in ion release (18). In contrast, our study did not exhibit such initial bursts, possibly due to differences in material composition or surface treatment. This absence of an initial burst aligns with other materials such as Cention N (19). The differences in initial ion release patterns across studies may stem from variations in polymerization protocols, filler compositions, and surface characteristics of bioactive materials. Gupta et al. noted that acidic environment facilitates better fluoride ion release as compared to neutral environment (11). The findings of our study are in general agreement with these reports. Kelic et al. note that it is more important to ensure sustained fluoride release over time than to achieve high initial bursts limited to a short period (20). Results of the current study indicate that release of fluoride ions met this requirement in both pre (Figure 1C) and post recharge (Figure 2C) settings. Overall, the findings of the current study are generally consistent with results reported in other ion-release studies (14, 18, 19, 21, 22).

Our study revealed notable differences in ion release between pre- and post-recharge phases. Specifically, the release of calcium, fluoride, and phosphate ions was significantly higher on earlier days compared to later days in both phases (Tables 1, 2, 4). Memarpour et al., in their study, reported a significant decrease in both calcium and phosphate ions from day 1 to day 3 (22). Our results reinforce this trend, suggesting that the early high release could be attributed to the initial surface dissolution of the material, which stabilizes over time. In the current study, the release of calcium, fluoride and phosphate were significantly higher on the earlier days as compared to later days in both the pre- and post-recharge phases (Tables 1, 2, 5). Referencing a study by Braga et al., Memarpour et al. explained that differences between studies could be attributed to variations in material composition, the type of acid used, and the methods employed for measuring ion release (21, 22). In our study, differences in ion release kinetics could also be linked to the resin matrix's interaction with the aqueous environment, which may modulate ion diffusion rates. Our study confirms that lower pH values, particularly between 4.5 and 5.5, are more conducive to ion release, especially on days close to charging. Nonetheless, the results presented unequivocally support the notion that lower pH values, particularly those between 4.5 and 5.5, are more conducive to calcium and phosphate ion release, especially on days close to charging (22).

Following the confirmation of “zero-ion” release, we subjected the samples to recharge with CPP-ACP paste (Tooth Mousse), recognized as a “smart material” for its pH-sensitive release of calcium and phosphate. As depicted in Figure 1 and Tables 3, 5, release of calcium, fluoride and phosphate were noticeable at pH 4.5, 5.5 and 6.5. There were minor differences between pH 4.5 and 5.5 for post-recharge ion release and studies with larger sample sizes may be needed to clearly ascertain the differential effects of lower pH ranges on ion release. In the current study, calcium ion release was measured with atomic spectrophotometry, fluoride and phosphate ions were measured using quantitative spectrophotometry. While other techniques such as chromatography and mass spectrometry could be used (23), spectrophotometric analysis was selected due to its wide dynamic range, high specificity, and sensitivity, making it a reliable method for characterizing ion release from biomaterials (24).

The choice of resin cement in this study was based on its bioactive properties and potential for remineralization, which are critical for evaluating its effectiveness in cariogenic conditions. The resin cement selected is known for its ability to release calcium, fluoride, and phosphate ions, which are essential for the remineralization process.

The “black box” of invitro studies is incontrovertibly different from “real-world” conditions and ion release at *in vivo* conditions may be influenced by many factors in the oral milieu and the composition of materials themselves. The results described herein come from one such “black box” and needs confirmation in additional clinical studies. For instance, in an *in vivo* setting, factors such as salivary flow, pellicle formation, and bacterial activity may significantly impact ion release patterns, making direct comparisons with *in vitro* studies challenging. *post-hoc* comparisons reported herein indicated that mean calcium and phosphate ion release showed statistically significant differences between pH 4.5 and 5.5 in a few instances. The limitations of this study include the small sample size, which may impact the generalizability of the findings. Additionally, our *in vitro* setup does not fully replicate clinical conditions. For example, in a clinical setting, the resin cement is typically covered by restoration, which can affect ion diffusion and release rates over time. Furthermore, in clinical practice, recharging resin cement would involve applying a fluoride-releasing agent or remineralizing paste beneath a prosthesis. This process is inherently more complex than *in vitro* conditions, as the recharging efficacy may depend on factors such as occlusal forces, pH fluctuations, and the permeability of the restorative material. Regular recharging protocols could be incorporated into routine dental care by recommending periodic application of remineralizing agents such as CPP-ACP or fluoride varnishes. Dental professionals could guide patients on at-home maintenance, ensuring optimal ion release and sustained remineralization. Additionally, incorporating recharging sessions in professional dental visits may enhance long-term benefits for high-risk patients. One key challenge in implementing recharging protocols is patient compliance. Adherence to regular application schedules may vary based on patient motivation, ease of use, and perceived benefits. Simplified application methods, patient education, and reinforcement strategies during dental visits could improve compliance and maximize the clinical effectiveness of bioactive resin cement.

## 5 Conclusion

In summary, the outcomes of this *in vitro* study indicate that bioactive resin cement demonstrates effective release of calcium, phosphate, and fluoride ions across both caries-safe and cariogenic pH levels. The substantial remineralization capacity observed at low pH suggests the potential to create a caries-safe environment, potentially enhancing the durability of indirect restorations.

However, given the limitations of invitro conditions, these findings require validation through extensive, long-term, randomized clinical trials. Future research should focus on evaluating ion release dynamics in real-time intraoral conditions, assessing the influence of saliva and biofilm on ion availability, and exploring potential surface modifications to optimize and prolong remineralization effects in clinical settings.

## Data Availability

The raw data supporting the conclusions of this article will be made available by the authors, without undue reservation.
