# Supplementary material for: Ion release dynamics of bioactive resin cement under variable pH conditions
Source: Front Oral Health. 2025 Mar 12;6:1564838. doi: 10.3389/froh.2025.1564838 (PMC11937140; doi:10.3389/froh.2025.1564838)
Supplement: Supplementary file 1 [file Supplementaryfile1.docx]

**Ion Release Dynamics of Bioactive Resin Cement Under Variable pH Conditions**

**Supplementary Information:**

**Supplemental Table 1:** Composition of Predicta Bioactive Resin Cement used in this study.

| **Predicta Bioactive Resin Cement base** | **Predicta Bioactive Resin Cement catalyst** |
| --- | --- |
| - Barium Glass and Zirconia filler (36%) - silica fillers (5%) - Calcium, phosphate and fluoride releasing fillers (20%) - Bis GMA (8%) - Urethane Dimethacrylate (11%) - Monomethacrylate and dimethacrylate monomers (16%) - Light cure and self-cure initiators, co-initiators and stabilizers (4%) | - Barium Glass filler (46%) - Silica fillers (3%) - Bis GMA (13%) - Monomethacrylate and dimethacrylate monomers (30%) - 4-META/10-MDP (4%) - Self-cure initiators and stabilizers (4%) |
| Uses: Cementation of indirect ceramic/composite/ metal crowns and bridges, inlays, onlays and endodontic posts. | |
